# Case Report: Oral lichenoid mucositis in a patient with metastatic renal cell carcinoma undergoing treatment with pembrolizumab and axitinib

**DOI:** 10.3389/fonc.2025.1495446

**Published:** 2025-05-19

**Authors:** Andrew Wellen, Michael J. Pierro, Karolyn A. Wanat, Ariel A. Nelson

**Affiliations:** ^1^ Department of Internal Medicine, Medical College of Wisconsin, Milwaukee, WI, United States; ^2^ Division of Hematology/Oncology, Department of Internal Medicine, Medical College of Wisconsin, Milwaukee, WI, United States; ^3^ Department of Dermatology, Medical College of Wisconsin, Milwaukee, WI, United States

**Keywords:** TKI toxicities, immune checkpoint inhibitor toxicities, lichenoid drug eruption, metastatic renal cell carcinoma (RCC), oral mucositis

## Abstract

Stomatitis is a relatively common adverse effect of systemic pharmacologic therapy seen in the treatment of various malignancies. When severe, stomatitis has the potential to limit a patient’s ability to tolerate optimal medical therapy and to impact quality of life. There are a variety of etiologies underlying stomatitis, including lichenoid drug eruptions. Herein, we present a novel case of a patient with metastatic renal cell carcinoma treated with pembrolizumab and axitinib who subsequently developed stomatitis consistent with a lichenoid drug reaction on biopsy. Pembrolizumab is known to cause such eruptions, and while there are no known lichenoid reactions reported for axitinib, stomatitis is one of the most common adverse effects of this therapy. In our case, it was not clear which systemic agent was the culprit. Axitinib was initially discontinued at the onset of symptoms. However, after a biopsy revealed lichenoid changes that are typically associated with pembrolizumab, pembrolizumab therapy was also stopped. With drug interruption and systemic glucocorticoid therapy, the patient’s stomatitis eventually improved. However, when axitinib was re-introduced to his treatment regimen, the patient experienced severe worsening of his oral lesions and development of new hand lesions requiring hospital admission. While the exact etiology of the lesions is unknown, the timeline of events appears to indicate a potential role of axitinib. This patient case represents a unique clinical scenario of axitinib contributing to a lichenoid drug eruption and highlights the importance of carefully considering the contributions of each agent in multidrug therapy regimens. Such cases are expected to become more frequent as combination immunotherapies and targeted therapies continue to gain approval across multiple solid tumor malignancies.

## Introduction

Stomatitis, or oral mucositis, is a common adverse effect of numerous cancer therapies that has the potential to decrease quality of life, limit oral intake, and contribute to weight loss. Additionally, significant stomatitis can promote non-adherence to treatment and may create interruptions in care that result in worsening of malignant disease (1). Stomatitis can arise through various mechanisms, and it is possible for a single medication to cause stomatitis through different pathways. Lichenoid drug eruptions (LDEs) are an example of an adverse effect that histopathologically and clinically resembles lichen planus (LP). Lichenoid drug eruptions are associated with many cancer therapies, though oral manifestations are comparatively rare (2–5). Under microscopy, a LDE is an interface dermatitis characterized by a band of lymphocyte infiltration at the dermal-epidermal junction and apoptosis of the basal keratinocytes (6). Clinically, papules are flat-topped, symmetrical, erythematous/violaceous, and oftentimes pruritic (7).

Pembrolizumab, a programmed cell death-1 (PD-1) inhibitor, is associated with a number of immune-related adverse events (irAEs), including cutaneous skin reactions such as morbilliform rash, LDE, and vitiligo; up to 40% of treated patients may potentially be affected by such dermatologic changes (8, 9). Cutaneous LDEs related to pembrolizumab have been reported in several cases (10–14). While oral complications of PD-1 inhibitors are less common than cutaneous reactions elsewhere in the body, cases of mucositis associated with pembrolizumab have been reported, including at least one case of oral erosive LDE (15–19).

Axitinib is a tyrosine kinase inhibitor (TKI) that targets the vascular endothelial growth factor (VEGF) pathway. It has been associated with a number of cutaneous adverse effects, commonly palmar-plantar erythrodysesthesia, or “hand-foot syndrome.” (20) While stomatitis is less common with axitinib compared to other TKIs, the reported incidence remains as high as 20% (21).

We present the case of a patient treated with both pembrolizumab and axitinib who developed oral lichenoid mucositis and highlight the complexities in identifying the etiology and management of such adverse reactions.

## Case

A 66-year-old man was diagnosed with International Metastatic Renal Cell Carcinoma Database Consortium (IMDC) poor risk, clear cell renal cell carcinoma (RCC) in November 2021 after presenting with a one-month history of right lower quadrant abdominal pain and hematuria. Staging workup revealed a 5.5 cm right renal mass, multiple pulmonary nodules, and a left parietal lobe intracranial lesion. Biopsies of the renal mass and lung were both consistent with clear cell RCC (cT1bcN0cM1). In December 2021, he presented with loss of consciousness and seizure activity secondary to the intracranial metastasis and underwent neurosurgical resection. He subsequently started systemic therapy with pembrolizumab (200mg IV every 21 days), followed by post-operative stereotactic radiosurgery in January 2022. Axitinib (5mg twice daily) was added to his treatment regimen one week after completing radiation at the end of January 2022.

**Figure 1:**
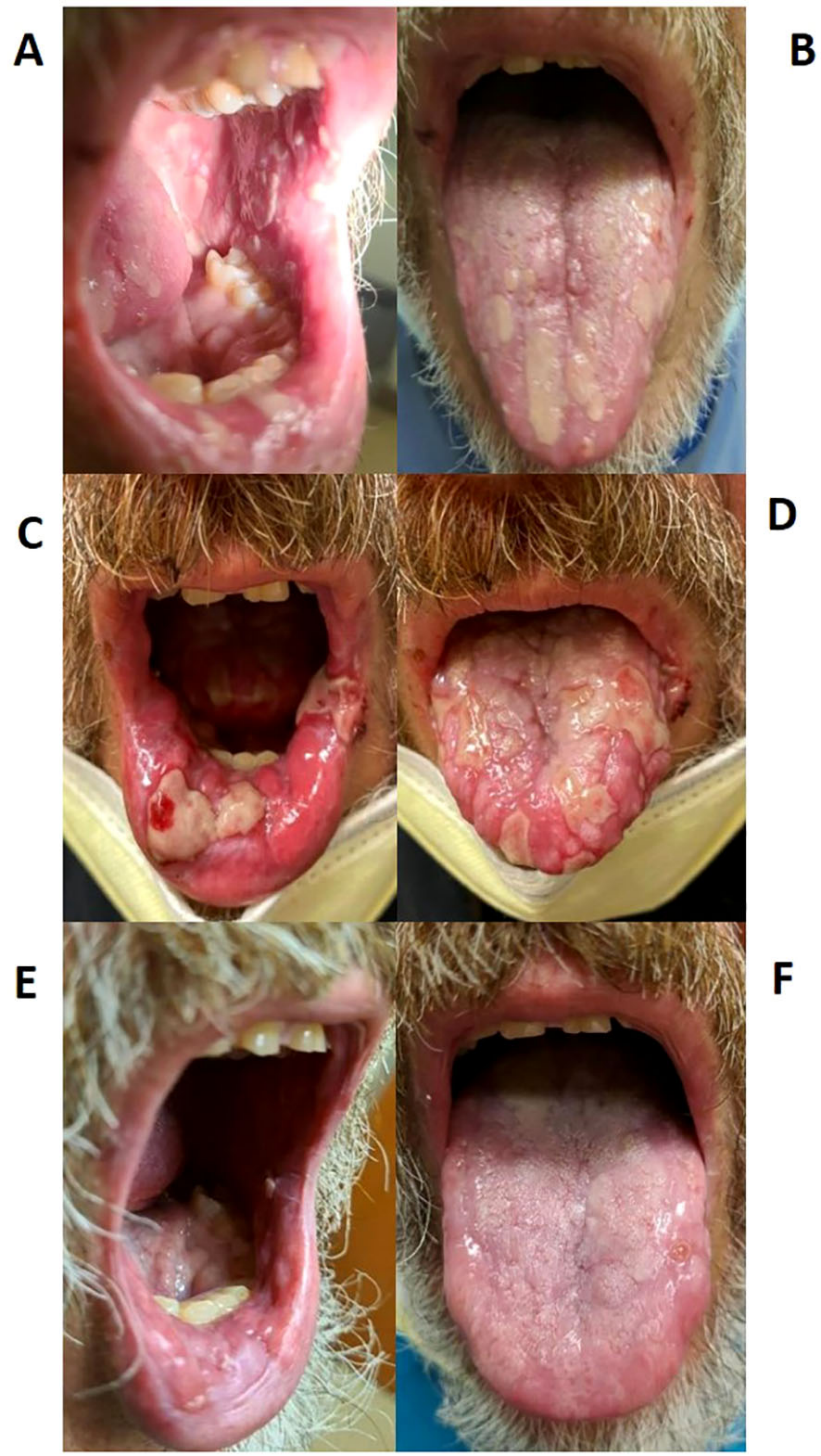
Stomatitis lesions over time. **(A, B)** Stomatitis at first diagnosis in March 2022; **(C, D)** Stomatitis during highest severity of disease in May 2022; **(E, F)** Stomatitis improvement after treatment in September 2022.

In March 2022, the patient noticed papules along the lining of his oral cavity, causing significant pain. Physical examination demonstrated eroded papules and plaques with scalloped borders and overlying fibrin located on his tongue, lips, and buccal mucosa, consistent with grade 2 mucositis (**Figures 1A, B**). Given the higher incidence of stomatitis with TKI therapy compared to PD-1 inhibitors, symptoms were attributed to the former, and axitinib was stopped. He was prescribed a mixture of oral anesthetic medications and oral dexamethasone swish, along with nystatin solution to cover a potential fungal etiology.

One week after stopping axitinib, the patient’s pain improved somewhat; however, the lesions continued to progress. He was then hospitalized for more intensive supportive care and an urgent dermatology consultation. The patient declined a biopsy of the lesions at that time. An initial swab for herpes simplex virus (HSV) was negative; however, a repeat swab was indeterminate, and he was started on empiric valacyclovir prior to hospital discharge.

**Figure 2:**
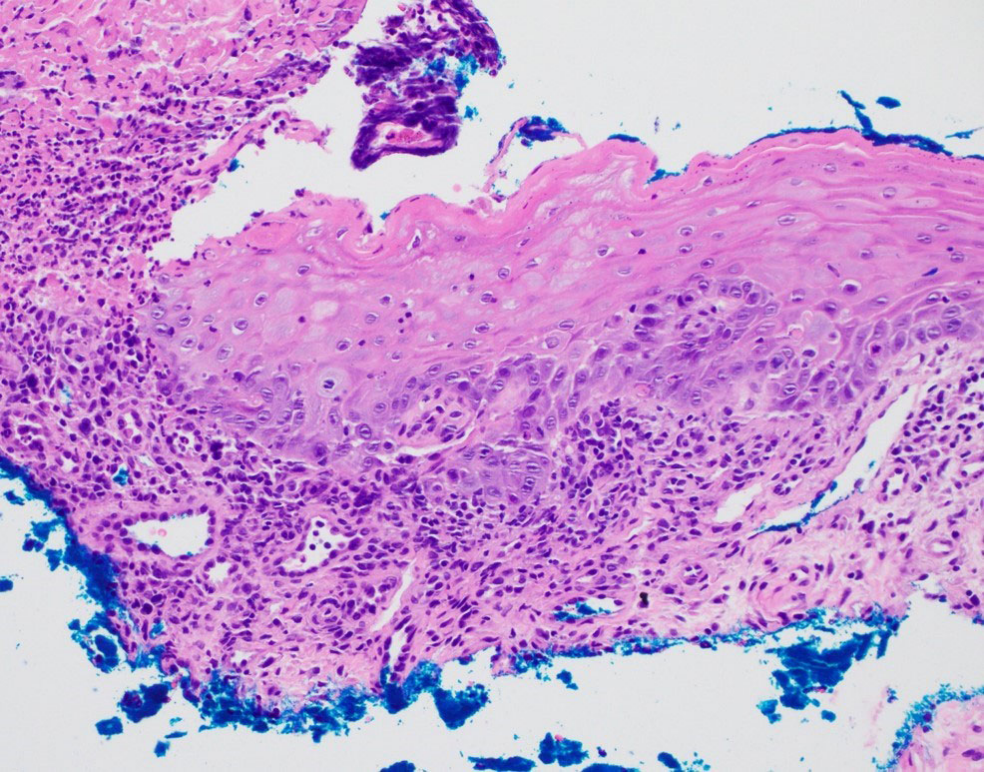
Histopathology demonstrates an ulceration with overlying crust and adjacent epithelium with a mild lichenoid infiltrate and focal dyskeratotic cells. No acantholysis was identified (hematoxylin and eosin staining, 20x original magnification).

Despite these interventions and a two-week pause of axitinib, the mucositis continued to worsen. Due to the persistence of his symptoms, a possible adverse effect of PD-1 inhibitor therapy was now considered. Pembrolizumab therapy was then stopped, and he was treated with oral prednisone (60mg daily). At this time, the patient agreed to punch biopsies of the lip and tongue lesions. Both biopsies demonstrated ulceration with adjacent mild lichenoid inflammation, consistent with LDE (**Figure 2**). Infectious stains (Gram, Grocott methenamine silver, and HSV) were negative.

After treatment with steroids and the discontinuation of both axitinib and pembrolizumab, the lesions started to improve, though did not completely resolve. In late April 2022, he was rechallenged with axitinib (5mg twice daily) while on prednisone (10mg daily). After one week of axitinib therapy, the lesions were no longer improving. His steroid dose was subsequently increased back to 20mg daily.

**Figure 3:**
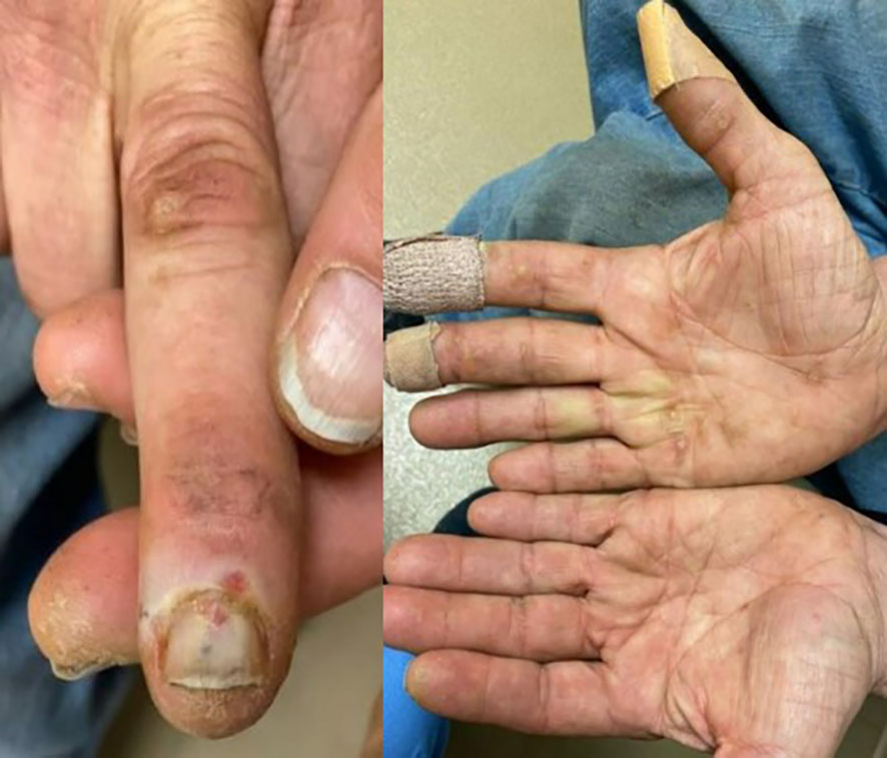
Hand lesions during highest severity of toxicity in May 2022.

After one month of axitinib with concomitant prednisone, the oral lesions continued to worsen along with newly developed painful ulcerations on his fingertips. Once again, axitinib was held. Over the following few weeks, the oral lesions worsened in number and severity, and the patient developed several new eroded and ulcerated papules on his bilateral palms and fingertips (**Figures 1C, D**, **3**). The symptoms necessitated hospital admission in mid-May 2022 and treatment with intravenous high-dose steroids, receiving two days of IV methylprednisolone (40mg twice daily).

**Figure 4:**
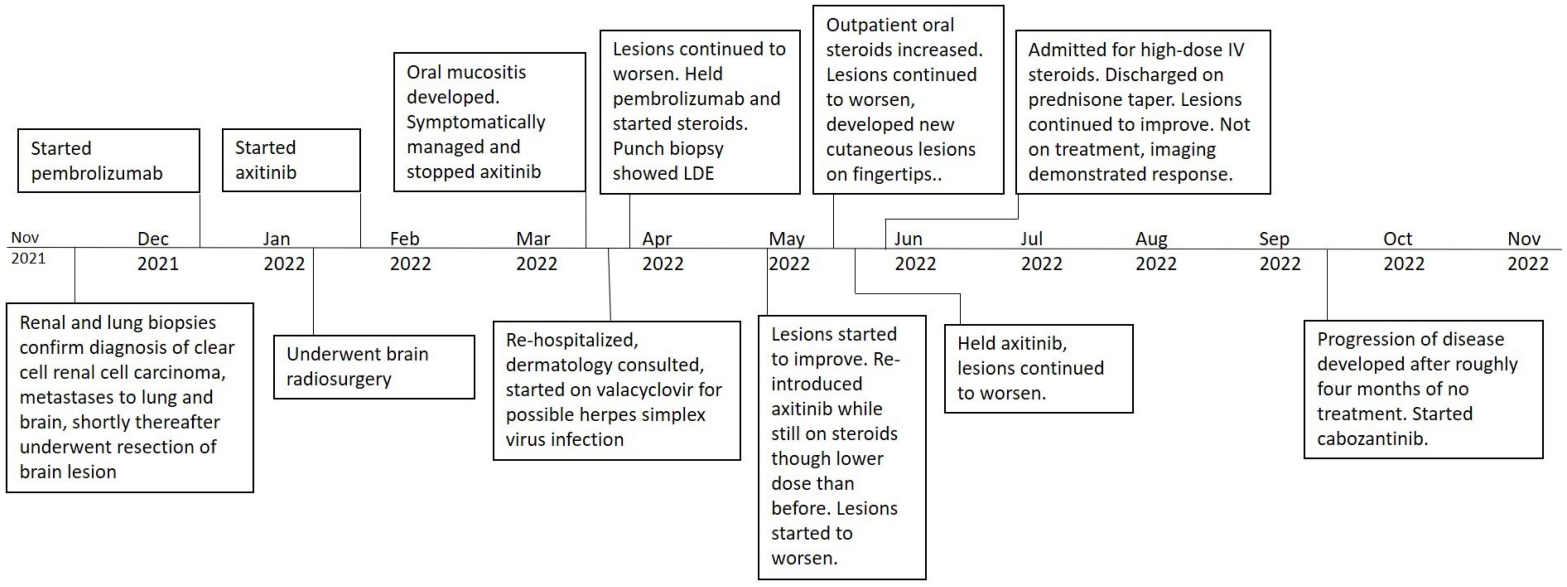
Disease course and toxicity timeline of events.

He was discharged on oral prednisone (60mg daily) until further in clinic follow-up two weeks later. Prednisone (40mg daily) with a weekly taper was then administered. The lesions continued to improve (**Figures 1E, F**). A disease-assessment CT scan demonstrated a partial response of his metastatic RCC lesions despite the interruptions in systemic therapy and steroid use. After a four-month treatment-free interval without progressive disease, he eventually developed progression of the known metastatic RCC lesions and was started on next-line therapy with cabozantinib (20mg daily) without any recurrence of oral LDE lesions (**Figure 4**). He continues cabozantinib, which has been titrated to 40mg daily, with an ongoing response in the metastatic RCC lesions as of the time of this publication.

## Discussion

This case highlights the complex interplay of different medications in the development of oral stomatitis. Pembrolizumab was initially thought to play the predominant role in this patient’s stomatitis, as the adverse reaction persisted and worsened until pembrolizumab was discontinued. Historically, PD-1 inhibitors have been more frequently associated with LDEs compared with VEGF TKIs. However, this patient developed worsening skin lesions when axitinib was reinitiated and pembrolizumab remained on hold. The mechanism by which the lesions worsened during axitinib treatment is not completely understood; here, we propose several potential explanations.

It is possible that the stomatitis was predominantly immunotherapy-mediated, and therefore worsened because of incomplete immunosuppressive treatment. This hypothesis is supported as the patient experienced clinical worsening during prednisone tapering and subsequent improvement with the resumption of higher-dose systemic steroids. However, the stomatitis worsened immediately after reinitiating the TKI, even after restarting a higher-dose oral steroid. Additionally, it had been improving on higher doses of the steroid taper before axitinib was reintroduced. Given these factors, it is likely that both medications contributed to the underlying process.

The mechanism by which stomatitis and hand-foot syndrome occur with axitinib is not well understood. However, it is plausible that axitinib’s anti-VEGF properties may impair the capillary network within the oral mucosa, thereby limiting steroid availability and hindering the migration of macrophages and other cells involved in healing pathways, ultimately worsening symptoms (22). It is also possible that axitinib instead triggered a separate, non-LDE effect on the oral mucosa and skin, and that the worsening oral lesions and new bilateral hand lesions were a distinct pathological process. As no further biopsies were pursued due to the patient’s preference, this possibility cannot be ruled out. However, we believe the development of an entirely new process was less likely, as the lesions happened concurrently and were clinically similar to the previously biopsied lesions.

When evaluating a patient experiencing an adverse effect of unknown etiology, maintaining a broad differential diagnosis is crucial. In this case, infectious etiologies were considered. Evaluation for herpetic infection was performed using both polymerase chain reaction and histology, with both ultimately yielding negative results. Other viral infections, such as CMV and EBV, as well as candidal infection, can also present with oral ulcerations; however, characteristic histopathological features of these infections were not observed, nor were organisms detected on staining. Furthermore, the patient’s response to steroids further reduced the likelihood of an infectious etiology.

Noninfectious etiologies, including paraneoplastic pemphigus (PNP), which can present with oral stomatitis in the setting of malignancy, were also considered. In such cases, indirect immunofluorescence on rat bladder epithelium, serological testing, ELISA, and immunoblotting may be useful diagnostic tools. However, histopathologically, there was no evidence of acantholysis on either biopsy in our case, which is often seen in PNP (23). Furthermore, several clinical factors did not support a diagnosis of PNP, including the resolution of the stomatitis with corticosteroids alone (as PNP is often recalcitrant to treatment), continued resolution after drug discontinuation despite the persistence of malignancy, and the absence of other mucosal involvement.

This patient case exemplifies a real-world, complex clinical scenario in which high-dose steroids and anti-cancer treatment discontinuation were required to resolve oral toxicity from first-line combination TKI and PD-1 inhibitor therapy, with both agents potentially contributing to the development of toxicity (24). The case also highlights the challenges of managing patients who may prefer fewer interventions, such as skin biopsies, and how these preferences can impact clinical decision making.

The optimal management of side effects from oncologic systemic therapy is often challenging. Clinical scenarios similar to ours are likely to become more frequent as combination therapy of TKIs and immune checkpoint inhibitors continue to gain approval across multiple solid tumor malignancies. As demonstrated by our report, these cases require a thorough history, careful physical examination, and often a multidisciplinary approach to ensure accurate diagnosis and optimal management.

We acknowledge the limitations of our report, including the lack of a follow-up biopsy and the inherent constraint of a single-case study. However, we present this case and our management strategy to raise clinician awareness and offer a potential approach for future cases. Greater familiarity with such cases may help deepen understanding of the mechanisms underlying toxicities in patients receiving concurrent immune checkpoint inhibitors and TKIs, which remain poorly understood.

## Data Availability

The original contributions presented in the study are included in the article/supplementary material. Further inquiries can be directed to the corresponding authors.
